# Turning over New Leaves

**Published:** 1888-08-18

**Authors:** 


### Turning Over New Leaves.

[Books for Review should be sent to The Editor, The Lodge, Porchester Square, W.]

### A Tale of the Unemployed.*

This book takes the form of a story, but no one who reads it can doubt that it is a narrative of bitter truth, told by one who knows the East-end, and knows those men whom, in newspaper phrase we class as "the unemployed," and he does not know them in mere journalistic fashion, to write sensational articles that will make a newspaper sell. Neither does he know them in the mere statistical fashion, that mentions as an interesting social fact that in the East-end of London alone 314,000 people are in almost daily danger of starvation. We know nothing of Mr. John Law, but one cannot read "Out of Work" without perceiving that it is written by one who knows individuals of this class, knows them in their poverty and their temptations, knows them in their vague clinging to the religion they have been brought up in, knows them in their sad seeking for better things outside the creeds and the churches, because they think if God is hard, no God may be kind. There is wit in the book, clever dialogue, shrewd character-sketching, but the power of it does not lie in these but in the fact that all it tells, all its terrible pictures of suffering, want, despair, and death are true, that these things are going on daily within a few miles of our own doors. The author draws no moral, says nothing of what the presence of this starving population may mean for the population that is not starving, that has, for the present at least, bread enough and to spare. But one cannot read this story, this simple narrative of a village carpenter who came to London seeking work and could not find it, who became a dock labourer, but at last failed to get employment even at the docks, and finally died of hunger "on tramp." One cannot read this without wondering if one day these hungry men and women will not rise up and demand food and clothing, and take it if the demand be refused. Admitting that the best of the working-classes get work, that our present system of competition does give "a fair field and no favour," one cannot but feel that for the second best, for those who could work even moderately well, this horrible doom of demoralising idleness and cruel hunger, is a thing which it is monstrous for any country to permit. It is easy to say that the poor are improvident, vicious, and impracticable; they may be all these things; but if the accusation were ten times truer than it is, it would form no excuse for those who profess to be the followers of One who came to call "not the righteous but sinners to repentance." If the rich do not accept the brotherhood of man as a fact that implies the paternal duty of help and sympathy—which mean more than mere almsgiving they can hardly expect the poor to recall it if a day of retribution comes.

To those who really want to know about the East-end and its inhabitants, but who have not time or courage to go "slumming," "Out of Work" may be recommended as a guide-book to the people of this unknown land. Here is the East-end sentiment regarding foreigners :—

"I was arguing with an old neighbour yesterday, a Christian. I asked him if he believed in hell.

"'Of course I do,' he said.

"'Shall you go there?' I wanted to know.

"'No,' he said.

"'Shall I?'

"'I hope not.'

"'Then who will?'

"'Them furriners,' he said; 'they'll go to hell.'"

Here again is a fact for those who like statistics :—

"It is reckoned that twenty thousand Londoners call themselves dock labourers, of which number nearly the half is daily turned away to swell the number of the unemployed, to seek, by fair means or foul, a living. Ten thousand! Just the number of that upper ten about which people are so fond of talking. A lowest ten, with wives and children."

Lastly, here is a picture of life at the docks which will startle even those who think they know something of how the poor live.

"Years hence, when children read in lesson-books about the Age of Competition, the docks will be given as an illustration of the competitive system after it reached a climax. Boys and girls will read that thousands of Englishmen fought daily at the dock gates for tickets; that starving men behind pressed so hard on starving men in front that the latter were nearly cut in two by the iron railings which kept them from work; that contractors were mauled by hungry men; that brick-bats and stones were hurled at labour-masters by men whose families were starving."

"Out of Work" is not fiction; it is history, and a history that should be widely read, in order that those who too rashly hope for the amelioration of the condition of the poor, and those who too rashly condemn the poor for their condition, may alike learn to think, and feel, and help.

* "Out of Work." By John Law. London: Swan Sonnenschein and Co.

### Fancy-Fair Religion.*

Mr. Priestly Foster has no sympathy with bazaars, fancy fairs, and other methods of raising money for charitable objects, which he stigmatises as attempts to serve God and Mammon, and looks upon as directly contrary to all Christian principle, trying to aid the cause of good by self-gratification instead of self-denial. We think that his strictures are rather too severe, and that he does not allow for the amount of real hard work and occasional self-denial that goes with the almost inevitable folly and vanity of bazaars of all kinds. Nevertheless, in view of the frequent quarrelling, heart-burning, and extravagance which mark these charitable sales, and the lack of even the morality of the gaming-table which is sometimes shown in conducting raffles, we must admit that they are not wholly undeserved. An instance which he quotes from a speech by Dr. A. K. H. Boyd at the opening of a Scotch bazaar is certainly a very bad specimen of bazaar swindling. "He had been lately informed," said the newspaper report, "of an instance in which the system followed appeared to him not fully justified. The subject raffled was a young pig. First of all tickets were sold amounting to £80—and he asked would any person lay his hand on his heart and say that any pig was worth £80? But there was something more behind that raffle, for he omitted to say that there was no pig at all. Everybody lost and nobody won, as the £80 was got for a pig which did not exist at all." This would certainly not be called fair dealing in an ordinary market, or even in an ordinary lottery, and some of the other ventures in connexion with fancy fairs, accounts of which Mr. Foster has collected, are as foreign to our notions of fitness as this to our notions of honesty, as for example the *tableaux vivants* in aid of a Roman Catholic charity, where "Two Strings to her Bow" was followed by "The Rock of Ages," both being considered "highly effective." Neither can one approve those adventitious methods of adding to the value of the wares which were more popular a few years ago than now. The incident Mr. Foster relates has been told before, but it is good enough to be worth repeating. "A simple young man was being served by a fascinating waitress with a cup of tea, the price of which he had been given to understand was sixpence. Before placing the said cup upon the table, the fair creature raised the cup of tea to her lips, and sipped the contents. The simple young man was too polite to do more than stare at what appeared to be the rather greedy behaviour of the amateur waitress, but he gave his 'thought no tongue,' and meekly tendered his sixpence. 'The cup of tea was sixpence before I sipped it; it is now half-a-guinea,' chirruped this maiden worker in a holy cause. 'Simple' manhood rose to the occasion, and with an irrepressible 'Oh! ah! hum! thank you! yes!' (ejaculations irrepressible under the circumstances), paid the 10*s.* 6*d.*, and quietly responded, 'And now will you kindly bring me a clean cup?'"

The temptation to raise money by means that do not appeal to our highest emotions is doubtless great, for all our charities seem to find a difficulty in raising funds by voluntary subscriptions. "It is a melancholy thought," Mr. Foster truly says, "that although everybody professes to be Christian, yet England finds an increasing difficulty in maintaining her hospital system. Lord Randolph Churchill has lately, when referring to the meagre support which modern society doles out to hospitals, asserted that "whereas the percentage of income derived from subscriptions in 1877 was 16·1, it has positively fallen in ten years to something like 14·4, which shows that there is a distinct falling-off of the efforts of the rich in support of these great institutions." Yet the help given by charity balls, bazaars, and the like is often marvellously small in comparison with the trouble and expense entailed in getting them up. One ball is quoted, a very successful ball, for which 800 guinea tickets were sold, of which sum every farthing was consumed in expenses, and all who have ever tried to raise money for charitable objects by similar means must recall the disappointment and vexation they felt in finding how small was the sum that remained for the cause they had at heart, after all the costs of the show, whatever it might be, had been deducted. Yet while people will not give spontaneously to a good object, we fear that all the scathing words that can be lavished on them will not sound the death-knell of fancy fairs. Money is wanted, and money will be raised by every means the law permits. Yet it is a pity that our people cannot be roused to a more lively appreciation of the words, "It is more blessed to give than to receive."

